# Population-specific Mutation Patterns in Breast Tumors from African American, European American, and Kenyan Patients

**DOI:** 10.1158/2767-9764.CRC-23-0165

**Published:** 2023-11-07

**Authors:** Wei Tang, Flora Zhang, Jung S. Byun, Tiffany H. Dorsey, Harris G. Yfantis, Anuoluwapo Ajao, Huaitian Liu, Margaret S. Pichardo, Catherine M. Pichardo, Alexandra R. Harris, Xiaohong R. Yang, Jonine D. Figueroa, Shahin Sayed, Francis W. Makokha, Stefan Ambs

**Affiliations:** 1 Laboratory of Human Carcinogenesis, Center for Cancer Research, NCI, NIH, Bethesda, Maryland.; 2 Data Science & Artificial Intelligence, R&D, AstraZeneca, Gaithersburg, Maryland.; 3 Colgate University, Hamilton, New York.; 4 Division of Intramural Research, National Institute of Minority Health and Health Disparities, NIH, Bethesda, Maryland.; 5 Department of Pathology, University of Maryland Medical Center and Veterans Affairs, Maryland Care System, Baltimore, Maryland.; 6 Department of Surgery, Hospital of the University of Pennsylvania, Penn Medicine, Philadelphia, Pennsylvania.; 7 Division of Cancer Control and Population Sciences, NCI, NIH, Rockville, Maryland.; 8 Integrative Tumor Epidemiology Branch, Division of Cancer Epidemiology and Genetics, NCI, Rockville, Maryland.; 9 Aga Khan University Hospital, Nairobi, Kenya.; 10 Mount Kenya University, Thika, Kenya.

## Abstract

Women of African descent have the highest breast cancer mortality in the United States and are more likely than women from other population groups to develop an aggressive disease. It remains uncertain to what extent breast cancer in Africa is reminiscent of breast cancer in African American or European American patients. Here, we performed whole-exome sequencing of genomic DNA from 191 breast tumor and non-cancerous adjacent tissue pairs obtained from 97 African American, 69 European American, 2 Asian American, and 23 Kenyan patients. Our analysis of the sequencing data revealed an elevated tumor mutational burden in both Kenyan and African American patients, when compared with European American patients. *TP53* mutations were most prevalent, particularly in African American patients, followed by *PIK3CA* mutations, which showed similar frequencies in European American, African American, and the Kenyan patients. Mutations targeting *TBX3* were confined to European Americans and those targeting the *FBXW7* tumor suppressor to African American patients whereas mutations in the *ARID1A* gene that are known to confer resistance to endocrine therapy were distinctively enriched among Kenyan patients. A Kyoto Encyclopedia of Genes and Genomes pathway analysis could link *FBXW7* mutations to an increased mitochondrial oxidative phosphorylation capacity in tumors carrying these mutations. Finally, Catalogue of Somatic Mutations in Cancer (COSMIC) mutational signatures in tumors correlated with the occurrence of driver mutations, immune cell profiles, and neighborhood deprivation with associations ranging from being mostly modest to occasionally robust. To conclude, we found mutational profiles that were different between these patient groups. The differences concentrated among genes with low mutation frequencies in breast cancer.

**Significance:**

The study describes differences in tumor mutational profiles between African American, European American, and Kenyan breast cancer patients. It also investigates how these profiles may relate to the tumor immune environment and the neighborhood environment in which the patients had residence. Finally, it describes an overrepresentation of *ARID1A* gene mutations in breast tumors of the Kenyan patients.

## Introduction

Breast cancer incidence rates vary substantially between geographic areas and population groups (1). The disease is the leading cause of cancer-related deaths among women worldwide (2). Women of African descent develop lethal breast cancer more frequently than women from other population groups in the United States, which is at least partly explained by disparities in access to care (3). They also have the highest occurrence of triple-negative tumors (4, 5). Currently, we do not know how much ancestral and neighborhood environmental factors contribute to aggressive breast cancer in these women but it has been shown that neighborhood deprivation may have an influence on breast cancer risk and survival and biological aging in patients with breast cancer (6–8). Investigations in West and Central Africa have provided corroboration that women of African ancestry tend to develop early-onset, high-grade, and estrogen receptor (ER)-negative tumors more frequently than women of European ancestry (9–12). However, these observations may not extend to East Africa (13, 14). Additional studies show that genetic predisposition could be an underlying cause for the high prevalence of ER-negative tumors among women of West African ancestry (15–18). We and others described additional traits of a distinct tumor biology in African American (AA) patients with breast cancer (19–24). More recently, these studies were enhanced with research into the mutational profiles of breast tumors of AA and indigenous African women from Nigeria (5, 11, 25, 26). Those investigations confirmed an elevated *TP53* mutation frequency in breast tumors of African descent women. In addition, they observed a potential deficiency in homologous recombination and an increased chromosomal instability in them. Despite these findings, it remains uncertain to what extent breast cancer in Sub-Saharan Africa beyond West Africa is reminiscent of breast cancer in AA or European American (EA) patients.

We therefore pursued the hypothesis that the mutational burden of breast tumors varies between population groups beyond current knowledge and may associate with the tumor immune environment and living in a deprived neighborhood. To test this hypothesis, we explored the mutational profile of breast tumors from AA, EA, and Kenyan patients using whole-exome sequencing (WES). We further investigated whether these profiles relate to the tumor immune environment and neighborhood deprivation, namely the neighborhood deprivation index (NDI), among the U.S.-based patients.

## Materials and Methods

### Tissue Collection

For the NCI-Maryland patient cohort, patients with breast cancer were recruited between 1993 and 2003, as described previously (27, 28). Additional patients were recruited at the University of Maryland (UMD) starting in 2012 as part of a study that will evaluate the impact of self-reported stress exposure on tumor biology. Of the 168 UMD patients included in the study, 9 were males. Patients with a family history of hereditary breast cancer were not part of this cohort. Race was self-reported as AA or Black and EA or White, with and without Hispanic ethnicity, or as Asian American. Patients completed a questionnaire and provided biospecimens at time of surgery. Samples of fresh-frozen tumor tissue and adjacent non-cancerous tissue were processed by a pathologist immediately after surgery at the Department of Pathology, UMD. Clinical and pathologic information was obtained from medical records and pathology reports. All patients provided written informed consent prior to tissue collection. Study protocols were approved by the UMD Institutional Review Board for the participating institutions (UMD protocol #0298229). The research was also reviewed and approved by the NIH Office of Human Subjects Research Protections (OHSRP #2248). For the Kenya patient cohort, tumor and adjacent non-cancerous tissue pairs were obtained from 23 women at the AIC Kijabe Hospital, Kijabe, and Aga Khan University Hospital, Nairobi, from 2019 to 2021. Following surgical excision, the collected tissue samples were immediately frozen in liquid nitrogen and stored at Aga Khan University Hospital. The tissues were then shipped on dry ice by air to the NCI in Bethesda, United States. Shipment took 3 days and the samples arrived embedded in dry ice. Patient information was abstracted from the medical records and included patient's age, tumor grade, tumor stage, and hormone receptor status, among others. All patients provided informed written consent prior to tissue collection and the study protocol was approved by the Research Ethics Committees (REC) at Aga Khan University Hospital (Ref: 2018/REC-80) and AIC Kijabe Hospital (KH IERC-02718/0036/2019). Permit to conduct the research was also sought from the National Commission for Science, Technology, and Innovation in Kenya. The research followed recognized ethical guidelines as defined by the Declaration of Helsinki and the U.S. Common Rule.

### WES

Genomic DNA was extracted from frozen breast tumors and adjacent non-cancerous mammary tissues (191 tissue pairs). Extractions were done with the Qiagen DNeasy blood & tissue kit, and the DNA quality was checked by the NIH Genomics Core using the Agilent Genomic DNA Screen tape assay. WES was performed by the service provider, Psomagen (https://www.psomagen.com/), which is Clinical Laboratory Improvement Amendments–certified and College of American Pathologists (CAP)-accredited, achieving a sequence depth of 250x for tumor tissues and 150x for adjacent non-cancerous tissues. A sequencing library was prepared by random fragmentation of the DNA or cDNA sample, followed by 5′ and 3′ adapter ligation. For a subset of samples, “tagmentation” that combines the fragmentation and ligation reaction into a single step was used because it greatly increases the efficiency of the library preparation process. Adapter-ligated fragments were PCR amplified and gel purified. For cluster generation, the library was loaded into a flow cell where fragments were captured on a lawn of surface-bound oligos complementary to the library adapters. Each fragment was then amplified into distinct, clonal clusters through bridge amplification. After completing the cluster generation, templates were ready for sequencing. Illumina sequencing by synthesis technology (https://www.illumina.com/science/technology/next-generation-sequencing/sequencing-technology.html) was utilized as a proprietary reversible terminator-based method that detects single bases as they are incorporated into DNA template strands. Using a single-base extension and competitive addition of nucleotides, single-base substitution (SBS) chemistry generates highly accurate sequencing data and virtually eliminates sequence-context-specific errors, even within repetitive sequence regions and homopolymers. An Illumina sequencer (NovaSeq 6000 system, RRID:SCR_016387) was used to generate raw images utilizing sequencing control software for system control and base calling through an integrated primary analysis software called RTA (Real Time Analysis, RRID:SCR_014332). The BCL (base calls) binary was converted into FASTQ utilizing Illumina package bcl2fastq (RRID:SCR_015058). The sequencing raw data and sample descriptors have been deposited in the Sequence Read Archive (SRA, RRID:SCR_004891) at https://www.ncbi.nlm.nih.gov/sra. The accession number for these data is PRJNA913947.

### Analysis of WES Data

FASTQ Read files were demultiplexed and trimmed for adapters and low-quality bases using Trimmomatic (RRID:SCR_011848) and then aligned to the human hg38 reference using the Burrows-Wheeler Aligner (BWA) software package (RRID:SCR_010910). Mapped reads were then deduplicated using Picard, followed by base quality score recalibration using the Genome Analysis Toolkit best practice workflow. Somatic variant calling was performed using MuTect2 in paired tumor-normal mode, and the panel of normal mode was enabled to reduce false positive discovers. Variants in VCF format were further annotated with functional and consequence prediction using Ensembl Variant Effect Predictor (RRID:SCR_007931) involving common databases and converted to Mutation Annotation Format (MAF) using the vcf2maf tool. MAF files of individual samples were concatenated into a combined MAF file and subject to a series of filtering steps, such as removing common variants with frequency larger than 0.001 in the ExAC, gnomAD, or 1000 Genomes in any specific subpopulations; removing variants with < 20× depth in the tumor sample; removing variants with < 5 reads of alternate allele, and an alternate allele frequency of less than 10%. Further quality check of variants involved manually displaying BAM files of variants in the Integrative Genomics Viewer (IGV) and retrieving mutation data from Catalogue of Somatic Mutations in Cancer (COSMIC; RRID:SCR_002260) and The Cancer Genome Atlas (TCGA; RRID:SCR_003193) BRCA MC3 mutation databases. The cleaned MAF file was then imported to MutSig2CV for driver mutated gene analysis. The R package maftools (RRID:SCR_024519) or in-house scripts were primarily used for data analysis and visualization such as generating oncoplots, mutually exclusive plots, lollipop plots etc.

### Tumor Mutational Burden

Tumor mutational burden (TMB) was calculated by summing up all the nonsynonymous somatic mutations including missense, nonsense, nonstop, frame shift deletions and insertions, in frame deletions and insertions, splice site and translations start site mutations detected in each of the patients. TMB was calculated per person with a capture region of 30 MB to obtain standardized frequency estimates.

### Mutational Signature Analysis, CIBERSORT, and NDI

Trinucleotide frequency patterns were extracted with maftools and the mutational signature analysis was performed with COSMIC V2. We compared individual trinucleotide patterns with single-base substitutions (SBS) mutational signatures in the COSMIC database that have been reported for breast cancer, namely SBS1–3, SBS5, SBS13, and SBS18. A mutational signature matrix was obtained for each subject. This matrix was used for group comparisons and related to either the tumor mutational spectrum, the tumor immune cell signature as defined by CIBERSORT (RRID:SCR_016955, https://cibersortx.stanford.edu/; ref. 29), or to neighborhood deprivation using either a correlation analysis or the Wilcoxon test to define the significance of group differences. To obtain neighborhood deprivation data for the UMD-based patients, their addresses were geocoded and linked to 1990 and 2000 census-tracts using the National Neighborhood Change Database (30). We defined neighborhood deprivation using an approach developed by Messer and colleagues (31). However, in deviation from the NDI developed by Messer and colleagues, which included 20 census variables, we prioritized a set of six socioeconomic status–related indicators, as described previously (32). The following variables are included in the index: percent households in poverty, percent female headed households with dependent children, percent households on public assistance, percent households earning under $30,000/year, percent males and females unemployed, and percent manager occupation. The index was standardized to have a mean of 0 and SD of 1. Lower values indicate lower deprivation, while higher values indicate higher deprivation. Finally, we performed a correlation analysis using deprivation indices derived from the 1990, 2000, and 2010 census-tracts and found they were highly correlated for our patient cohort (correlation coefficient *r* > 0.9 for all comparisons), indicating that working with an additional NDI derived from 2010 census-tract data would not be required for our study.

### RNA-sequencing Data Analysis

RNA isolated from 68 frozen tumors was sent to the NCI Center for Cancer Research Sequencing Facility for library preparation with the TruSeq PolyA kit (Illumina). Sequencing was performed with the NovaSeq system using 150 bp paired-end reads with a sequence depth of at least 30 million reads. Reads were trimmed with the Trimmomatic software with 90% of them being uniquely aligned to the human genome (hg38) using STAR (RRID:SCR_004463). RNA mapping statistics were calculated using Picard with more than 90% of the reads being mapped to the transcriptome. Read count per gene was calculated by HTSeq (RRID:SCR_005514) under the annotation of Gencode (RRID:SCR_014966) and normalized by size factor implemented in the DESeq2 package. Regularized-logarithm transformation (rlog) values of gene expression were used to perform further analyses. The RNA sequencing (RNA-seq) data were deposited in the NCBI's Gene Expression Omnibus (GEO) database (RRID:SCR_005012) under accession number GSE225846.

### Pathway Enrichment

#### By Mutation Status

Transcriptome data from TCGA human breast dataset were subjected to a gene set enrichment analysis (GSEA, RRID:SCR_003199). GSEA was performed as described previously (33). For pathway enrichment analysis, genes were ranked by t-statistic and imported into the GSEA preranked module (https://software.broadinstitute.org/gsea/index.jsp;). Kyoto Encyclopedia of Genes and Genomes (KEGG) gene sets (*n* = 186) were selected within MSigDB (RRID:SCR_016863) as references for the pathway analysis.

#### By Population Group

Differential expression analysis was done using DESeq2 package in R (RRID:SCR_015687). Population group-specific differentially expressed genes (DEG) were filtered by a *q* value (FDR) < 0.05, the absolute value of fold change > 2. We then performed pathway enrichment analysis with DEGs upregulated in AA breast tumors (compared with Kenyan and EA breast tumors) through overrepresentation analysis using canonical pathways (MSigDb, Broad Institute). Pathways with an FDR < 0.05 were used for subsequent biologic interpretation.

### Single-sample GSEA

Pathway activity scores derived from single-sample GSEA were calculated with the Gene Set Variation Analysis (GSVA) R package (ref. 34; RRID:SCR_021058), it provides an estimate of pathway activity by transforming an input gene-by-sample expression data matrix into a corresponding gene-set-by-sample expression data matrix. KEGG pathways (RRID:SCR_012773) were used as the reference gene sets. We chose the z-score method implemented within the GSVA package to represent the activity in each sample. To calculate a pathway activity score for oxidative phosphorylation, we selected the gene expression profile for the genes annotated in KEGG “oxidative phosphorylation” and summed this profile into a z-score as the pathway activity score for each tumor.

### Ancestry Estimation

We estimated genetic ancestry using the WES data from the tumor/normal tissue pairs. As a first step, germline variants were called using GATK's HaplotypeCaller in joint genotyping mode. Variants were then filtered for quality with the following criteria: QD < 2.0, FS > 60.0, MQ < 40.0, MQRankSum < −12.5, ReadPosRankSum < −8.0 for SNPs; QD < 2.0, FS > 200.0, ReadPosRankSum < −20.0 for INDELs. For admixture analysis, only SNPs that were biallelic were retained in the analysis. VCF files were converted into PLINK format to calculate the distance matrix. 1-(identity-by-state) was used as implemented in PLINK with “—distance” function. We then used GRAF-pop (https://www.ncbi.nlm.nih.gov/projects/gap/cgi-bin/Software.cgi) which is a fast distance-based method to infer ancestry based on references from multiple genotype datasets, including those of populations of Caucasian, African, AA, Asian, and Mexican descent.

### Statistical Analysis

All statistical tests were two sided, and an association was considered statistically significant at *P* < 0.05. For survival analysis, we used either the Kaplan–Meier method, together with a log-rank test for significance testing, or Cox regression modeling. For the NCI-Maryland patient cohort, we had National Death Index–based survival follow up for 80 AA and 64 EA patients, including the 9 males, through December 31, 2020. Statistical analyses were performed using the R software, and the packages in Bioconductor (RRID:SCR_006442, https://www.r-project.org) provided by the R Foundation for Statistical Computing.

### Data Availability

The data generated in this study are publicly available. The WES raw data for the 191 tumor/adjacent normal tissue pairs and sample descriptors have been deposited in the SRA at https://www.ncbi.nlm.nih.gov/sra. The accession number for these data is PRJNA913947. RNA-seq data for 68 human breast tumors with paired WES data were deposited in the NCBI's GEO database under accession number GSE225846. Additional information can be obtained from the corresponding author, Stefan Ambs, upon request.

## Results

### Study Design

**FIGURE 1:**
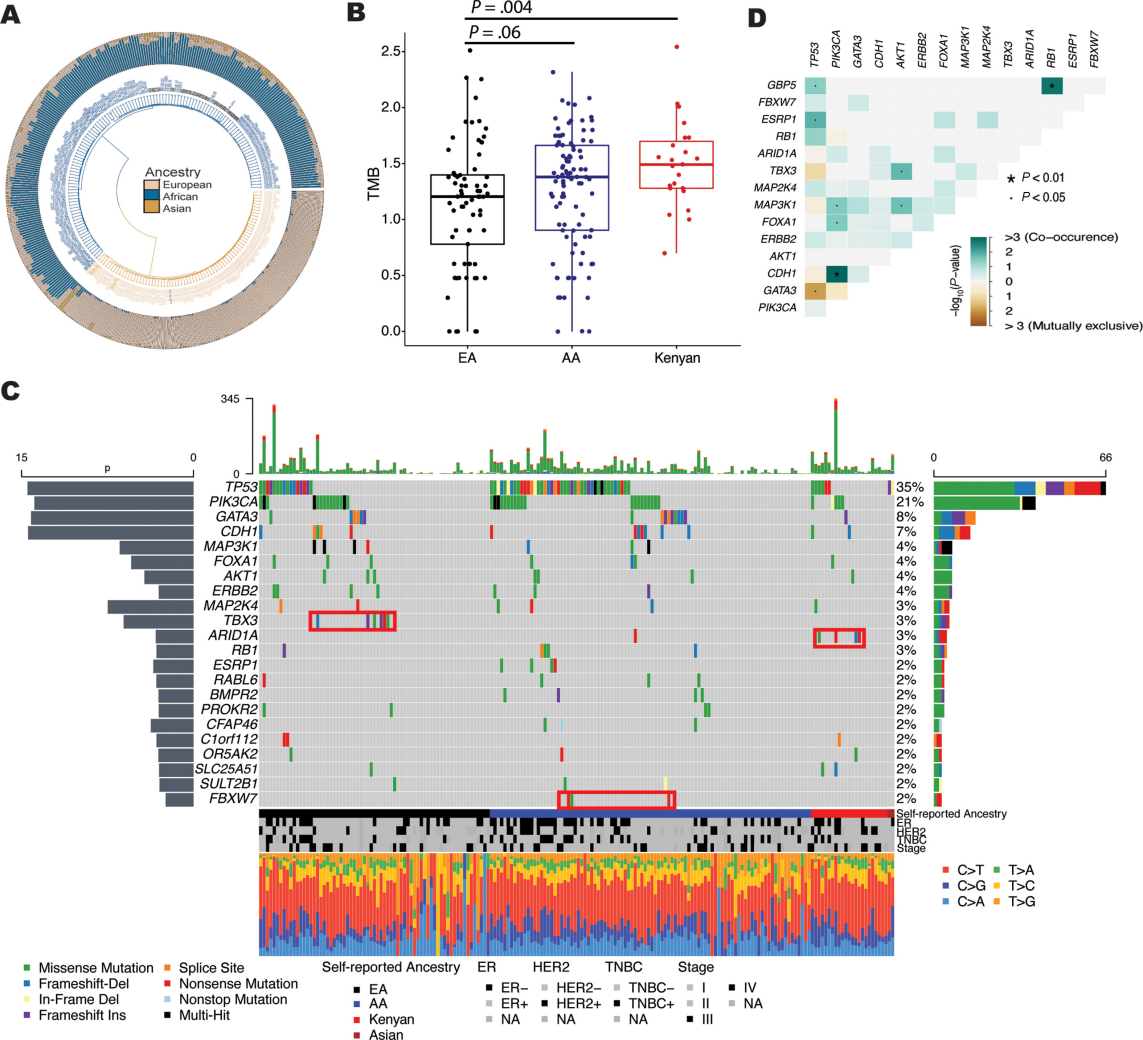
Mutational profiles in breast tumors according to ancestral background of the patients. **A,** Circos admixture plot showing proportions of West African, European, and Asian ancestry (outer circle) in self-identified AA (blue, inner circle, *n* = 97), EA (bisque, inner circle, *n* = 69), and Asian American (bronze, inner circle, *n* = 2) patients with breast cancer in the NCI-Maryland study. Circos plot shows ancestry estimates derived from tumor/normal tissue pairs. **B,** TMB in primary breast tumors of EA (*n* = 69), AA (*n* = 96), and Kenyan patients with breast cancer (*n* = 23). TMB as a standardized measure (log_10_ scale) is different between EA, AA, and Kenyan patients (one-way ANOVA test, *P* < 0.05), and is highest in Kenyan tumors. **C,** Oncoprint plot showing all somatic mutations in candidate driver genes that occurred at a frequency ≥2% in 191 primary breast tumors, ordered by patient group and mutation frequency. Red boxes highlight mutations that occurred at an increased frequency in either EA (black bar beneath plot, *n* = 69), AA (blue bar, *n* = 97), or Kenyan (red bar, *n* = 23) patients. The color scheme of the bars within the plot indicates the mutation type and is explained beneath the plot to the left. The green and red color bars at the top of the plot summarize all somatic mutations detected within one tumor, with green and red indicating the predominance of missense and nonsense mutations among all mutations. The gray bar to the left indicates significance as −log_10_(*P* value) and was generated by the MutSigCV software, identifying excessively mutated genes. The more the bar extends, the more likely it is that the mutated gene is cancer-related driver gene. **D,** Correlation matrix (*P* value-based) of mutual exclusivity among the detected mutations and their target genes. As examples, *GATA3* and *TP53* mutations did not co-occur but *PIK3CA* and *CDH1* mutations commonly did.

We generated somatic mutation profiles for breast tumors from 168 U.S.-based patients, inclusive of 9 male patients (Supplementary Table S1), and S23 Kenyan patients (Supplementary Table S2) by interrogating WES data generated from both tumor tissue and paired adjacent non-cancerous tissue. The Kenyan patients with breast cancer tended to be noticeably younger than the U.S. patients with breast cancer [mean age: 48.8 (Kenyan) vs. 56.8 (AA) vs. 56.6 (EA) years]. The three patient cohorts had a similar body mass index (BMI) distribution with most patients being in the overweight to obese categories, according to World Health Organization criteria. Also, more patients presented with ER-positive than ER-negative disease in each cohort (70% Kenyan; 59% AA, 72% EA). All but one of the AA patients had West-African ancestry estimates exceeding 50%, whereas European ancestry predominated in all self-identified European-American patients (Fig. 1A).

### TMB

**FIGURE 2:**
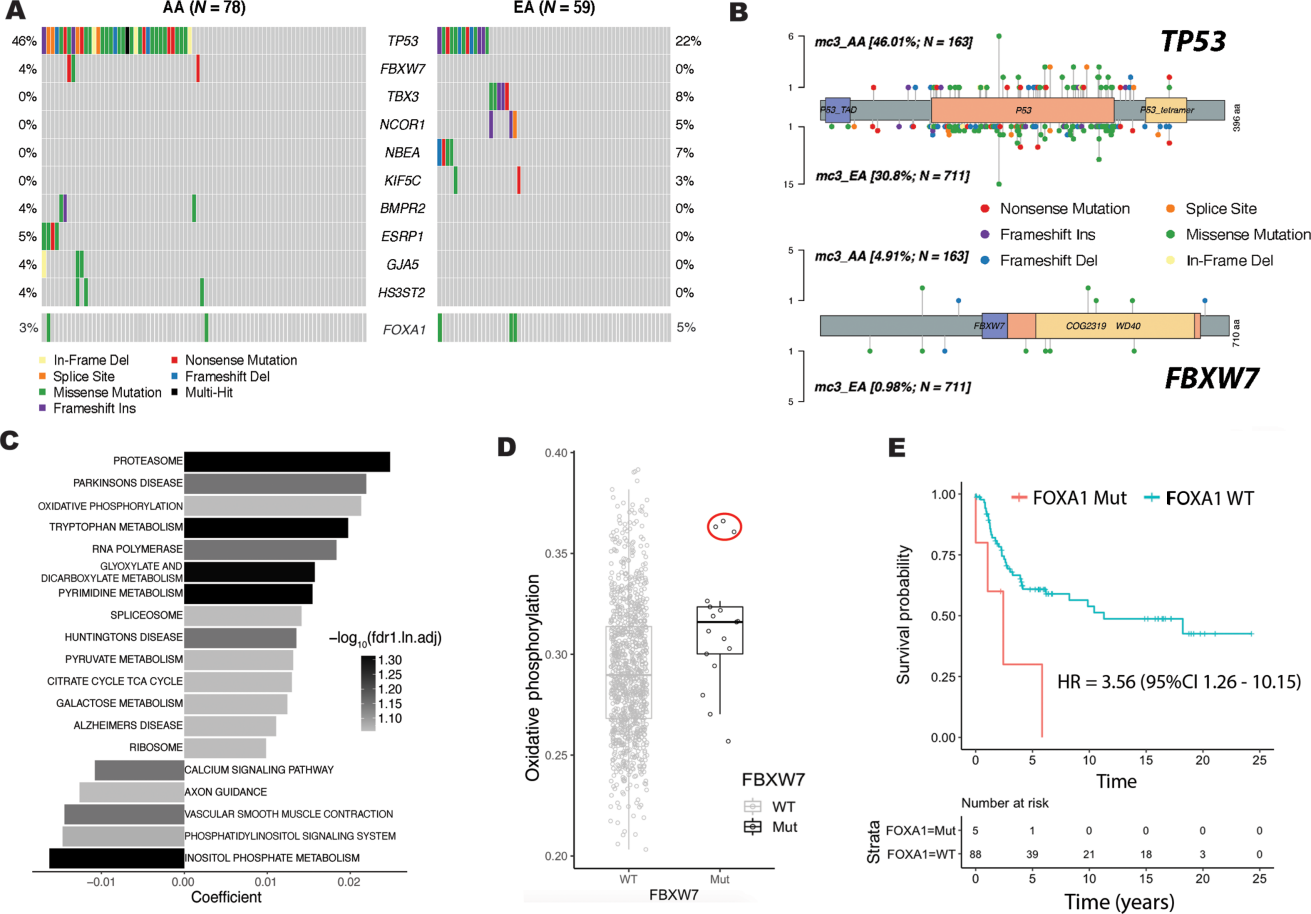
Pattern of somatic mutations among AA and EA patients. **A,** Genes enriched with somatic mutations showing robust frequency differences or exclusivity in AA and EA patients with breast cancer. *FOXA1* mutation frequency is shown as comparison. Only those patients who carried at least one of these mutations are included in this graph. **B,** Mutational spectra of *TP53* (top) and *FBXW7* (bottom) tumor suppressor genes in the AA and EA patients in TCGA dataset. **C,** GSEA KEGG gene set pathways with either positive or negative enrichment of DEGs comparing the transcriptome of *FBXW7* mutant versus *FBXW7* wild-type breast tumors. FDR ≤ 10%, with adjustment for patients’ race. Source: TCGA breast cancer dataset. **D,** Oxidative phosphorylation capacity in breast tumors with (*n* = 17) and without *FBXW7* mutations (*n* = 1,046). Tumors carrying *FBXW7* mutations have a significantly higher predicted capacity (Wilcoxon rank-sum test, *P* < 0.05). Oxidative phosphorylation capacity was derived from a meta-gene score covering expression of genes in the KEGG oxidative phosphorylation pathway. Red circle: tumors carrying the R465C *FBXW7* mutation. Oxidative phosphorylation pathway activity scores were obtained from single-sample GSEA. Source: TCGA breast cancer dataset. **E,** Mutations in the *FOXA1* gene have the most deleterious effect on survival of patients with breast cancer in the NCI-Maryland breast cancer cohort (*n* = 93). Kaplan–Meier plot with a HR estimate using Cox regression modeling.

It has been previously reported that tumors from patients of African ancestry may show a homologous repair deficiency and a generally increased somatic mutation burden (11, 35). We examined the TMB in the three patient groups and found—based on the somatic mutation frequency in the WES-defined genome—that the TMB was highest in the Kenyan samples, lower in AA, and lowest in the genome of breast tumors from EA patients (Fig. 1B). Next, we investigated the mutational profiles of the 191 breast tumors from the U.S. and Kenyan patients, focusing on candidate driver mutations affecting protein-coding genes at a frequency of ≥2% across all patients. As shown by the oncoplot (Fig. 1C), *TP53* mutations were the most common mutational event (35%) with the highest frequency in AA (43%), followed by Kenyan patients (35%), and the lowest frequency in EA patients (23%). *PIK3CA* mutations were the second most common alteration, consistent with the literature (5, 36), but with similar frequencies in EA (20%), AA (21%), and Kenyan patients (22%) in this cohort. Among other genes with lower mutation frequencies, some stood out: *TBX3* being mutated only in EA patients (9%, *n* = 6), ESRP1 and *FBXW7* only in AA [4% (*n* = 4) and 3% (*n* = 3), respectively], and *ARID1A* being most frequently mutated in Kenyan patients (17%, *n* = 4). A correlation analysis revealed that *GATA3* and *TBX3* mutations both inversely correlated with *TP53* mutations. Their occurrence and the occurrence of a *TP53* mutation were exclusive from one another. In contrast, *RB1* co-occurred with *GBP5* and *CDH1* with *PIK3CA* mutations (Fig. 1D). In an analysis restricted to AA and EA patients, several mutations showed associations with the patient group and the tumor *TP53* mutational status (Fig. 2A). Among them, *ESRP1* mutations occurred only in AA patients carrying a *TP53* mutation and *NBEA* mutations occurred only in EA patients with a *TP53* mutation. Conversely, *TBX3* and *NCOR1* mutations occurred exclusively in EA patients who did not carry a *TP53* mutation in their tumors.

*FBXW7* encodes an E3 ubiquitin ligase complex member and tumor suppressor (37). Like *TP53* mutations, inactivating *FBXW7* mutations occur throughout the gene body in human breast tumors (Fig. 2B). These mutations are increased in patients with cancer of African descent, showing a consistent association with West African ancestry, as reported previously (38). We observed these mutations in AA patients, but not in EA or Kenyan patients, in our cohort. We confirmed their increased occurrence among AA patients in TCGA breast cancer dataset (AA: 4.91%; EA: 0.98%; Fig. 2B). To further understand the impact of *FBXW7* mutations on breast cancer biology, we performed a GSEA using RNA-seq data and KEGG pathway annotation for the contrast *FBXW7*-mutant versus wild-type tumors (Fig. 2C). For statistical power, we performed this analysis within TCGA breast cancer dataset, harboring 17 tumors with *FBXW7* mutations, but not in our cohort because only 3 among the 191 patients were carriers of a *FBXW7* mutation. Enriched pathways for this contrast (mutant vs. wild-type *FBXW7*) included the proteosome pathway as the top ranked pathway. The observation agrees with the known function of FBXW7 as an ubiquitin-proteosome ligase involved in the degradation of putative oncogenic proteins. Other enriched pathways included the process of mitochondrial oxidative phosphorylation (Fig. 2C). We therefore explored whether the gene set–based pathway z-score for KEGG-defined oxidative phosphorylation is higher in *FBXW7*-mutant when compared with wild-type tumors. This analysis showed that *FBXW7*-mutant patient tumors, especially those with a R465C mutation (3 AA patients), had significantly increased pathway activity scores when compared to *FBXW7* wild-type breast tumors (Fig. 2D), indicating increased mitochondrial activity in *FBXW7*-mutant tumors. We did not find that patients with breast cancer with *FBXW7*-mutant tumors had a significantly different survival than patients with wild-type tumors, in part because only few patients carried such a mutation. In contrast, somatic mutations in the *FOXA1* gene robustly associated with decreased patient survival in both our (Fig. 2E) and TCGA-Broad GDAC breast cancer cohort (Supplementary Fig. S1). Mutations in this gene also occurred at a relatively low frequency (2%–3%). Yet, their occurrence robustly predicted breast cancer lethality.

Because we could not investigate gene expression profiles associated with infrequent mutations in our own dataset (e.g., for the *FBXW7* and *ARID1A* genes), we compared differential gene expression by population group and focused on pathway enrichment of DEGs. We report the DEGs in Supplementary Table S3. As the key observation, pathways related to oxidative phosphorylation were significantly enriched in tumors from AA patients relative to tumors from both Kenyan (electron transport chain: oxidative phosphorylation system in mitochondria, FDR = 1.77E-7) and EA (oxidation by cytochrome *P450*, FDR = 2.1E-2) patients, indicating a distinct pathway activation in AA patients when compared with the other patient groups. These findings are consistent with a previous report showing a pan-cancer upregulation of mitochondrial oxidative phosphorylation in tumors of AA patients, when compared with EA patients (39).

**FIGURE 3:**
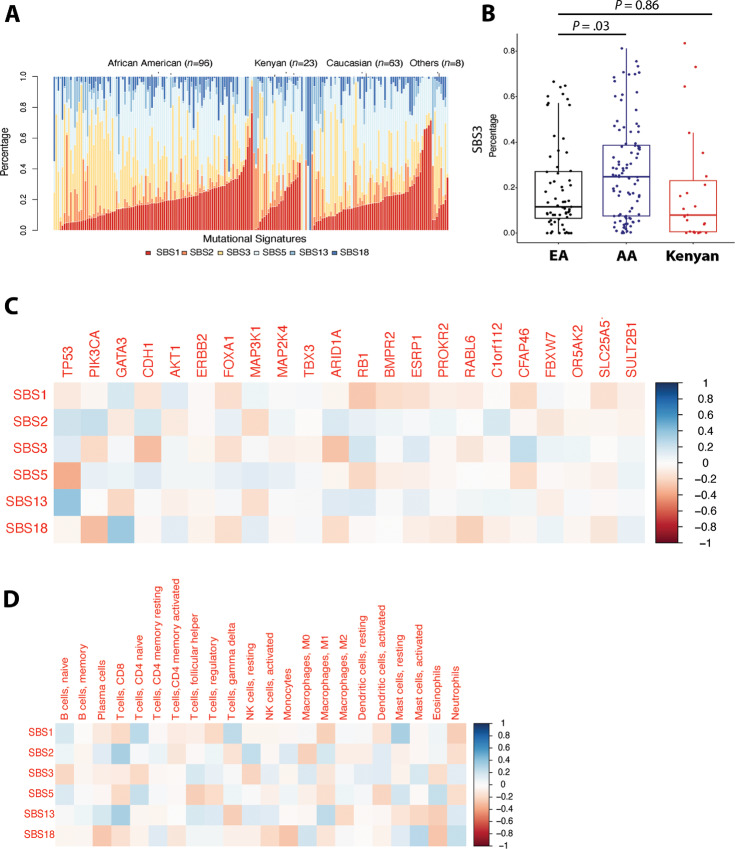
Mutational signatures in breast tumors and their association with somatic mutations in driver genes and immune cell signatures. **A,** Prevalence of mutational signatures from the COSMIC catalog (SBS1–3, 5, 13, 18) in breast tumors from AA, Kenyan, and EA patients. Others include 2 AA and 6 EA patients with self-reported Hispanic ethnicity. **B,** Elevated presence of the SBS3 mutational signature in breast tumors of AA patients. Relative abundance scores for SBS3 in each tumor were compared between the three patient group patients (one-way ANOVA, *P* < 0.05). **C,** Heat map showing a correlation coefficient matrix for the relationship between the six COSMIC-based mutational signatures and somatic mutations in candidate driver genes in 191 breast tumors. **D,** Heat map showing a correlation coefficient matrix for the relationship between the mutational signatures and gene expression–based immune cell profiles of the tumors. Transcriptome data and the CIBERSORT algorithm were used to define the immune cell profiles.

After examining the frequency of candidate driver mutations, we asked whether breast tumors may show significant differences among the three patient groups in harboring previously defined mutational signatures. We retrieved these signatures from the COSMIC (40) and then compared their frequencies. We focused on six mutational signatures that are prevalent in breast cancer, namely SBS 1–3, 5, 13, and 18 (41, 42). We confirmed their presence (Fig. 3A) and compared their frequency across the patient groups. One signature showed variance. The SBS signature 3, SBS3, occurred at a higher frequency in AA but not Kenyan patients when compared with EA patients (*P* = 0.03, AA vs. EA; *P* = 0.86, Kenyan vs. EA; Fig. 3B). An elevated frequency of SBS3, which indicates a defective homologous recombination–based DNA repair pathway, has previously been described for breast tumors from AA and Nigerian patients (11, 41). We did not find that the occurrence of SBS3 was significantly correlated with a particular driver mutation in the same tumors (Fig. 3C). Somatic or germline mutations in *BRCA1/2* that can cause a SBS3 signature did not occur in our patient cohort. In contrast, SBS5 showed an inverse correlation with the occurrence of *TP53* mutations (correlation coefficient: −0.36) whereas SBS13 and SBS18 positively correlated with the occurrence of either *TP53* (correlation coefficient: 0.38) or *GATA3* mutations (correlation coefficient: 0.36; Fig. 3C). SBS18 is an oxy-radical damage signature that was reported to be increased in Chinese patients with breast cancer (43).

### Tumor Mutation Signatures and Immune Profile

Next, we assessed the association of these mutational signatures with gene expression–based immune cell profiles using the CIBERSORT deconvolution algorithm (29). We restricted this analysis to the 68 breast tumors with both WES and transcriptome data (27 AA, 18 EA, 23 Kenyan patients). T follicular helper cells and uncommitted macrophages (M0) showed variance among the patient groups and were increased in AA but not Kenyan patients, when compared with EA patients (Supplementary Fig. S2). Yet, the differences were not significant in a multicomparison-adjusted analysis across all CIBERSORT contrasts. We further noticed positive associations between the number of tumor-infiltrating CD8-positive T cells and the SBS2 and SBS13 signatures (correlation coefficient 0.33 and 0.32, respectively; *P* < 0.05 each; Fig. 3D). Yet overall few of these relationships existed with correlation coefficients ≥0.3 (Fig. 3D). SBS2 and SBS13 commonly co-occur in tumors and are attributed to an upregulated AID/apolipoprotein B mRNA editing enzyme, catalytic polypeptide-like (APOBEC) cytidine deaminase activity in tumors (https://cancer.sanger.ac.uk/signatures/sbs/). We performed an additional sensitivity analysis with male and Asian American patients being removed from the dataset (Supplementary Fig. S3). We found that our results remained largely unchanged, with SBS3 occurring at the highest frequency in AA patients and the number of tumor-infiltrating CD8-positive T cells being associated with the SBS2 and SBS13 signatures (correlation coefficient 0.31 and 0.35, respectively; *P* < 0.05 each).

### Tumor Mutation Signatures and Neighborhood Deprivation

**FIGURE 4:**
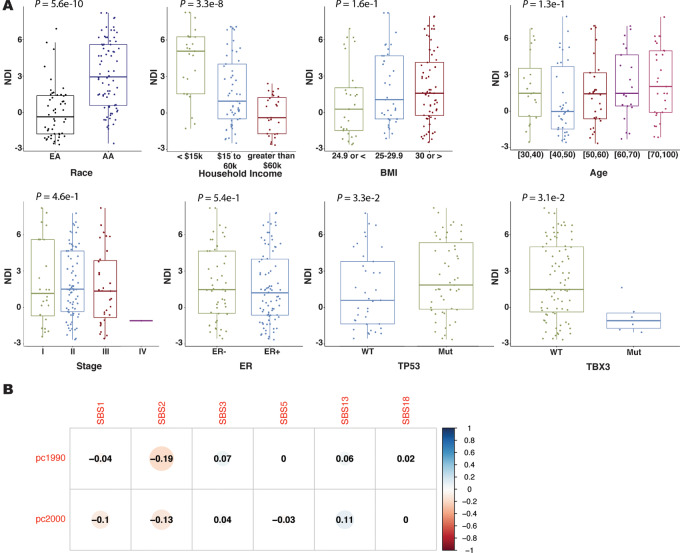
Neighborhood deprivation and its association with mutational signatures. **A,** Relationship of the NDI with race/ethnicity, household income, BMI, age, disease stage, tumor ER status, and tumor *TP53* and *TBX3* mutational status, in the NCI-Maryland breast cancer cohort. NDI for the analysis was obtained for each patient in the study using 2000 census data and correlated with patient or tumor characteristics. Significant associations with race, household income, and *TP53* and *TBX3* mutational status (*P* < 0.05 with *t* test or one-way ANOVA). **B,** Correlation matrix for the relationship of patients’ 1990 and 2000 NDIs with six COSMIC-based mutational signatures in their tumors. NDI shows a moderate inverse correlation with the SBS2 signature.

Neighborhood factors may influence tumor biology. We inquired whether the neighborhood environment may associate with the occurrence of these mutational signatures in our U.S. cohort. To do so, we generated a NDI for all U.S. patients, as described previously (32). Comparable NDI data did not exist for the Kenyan patients. Because our U.S. patients were recruited between 1993 and 2004, we applied NDIs calculated with both 1990 and 2000 census data. Neighborhood deprivation significantly associated with patient survival independent of other known survival predictors including a patient's income and education, self-reported race, or disease stage, with an estimated 30% proportion as an independent mediator in predicting survival outcomes in the NCI-Maryland cohort (Supplementary Fig. S4A–S4C). It also associated with patient and disease characteristics (Fig. 4A) but did not correlate significantly with any of the mutational signatures (Fig. 4B). In a sensitivity analysis with male and Asian American patients being removed from the dataset, the associations of NDI with patient and disease characteristics (self-reported race, household income, and *TP53* and *TBX3* mutational status) remained (Supplementary Fig. S5).

## Discussion

There is evidence that West African ancestry may influence breast cancer biology (11, 18, 19, 44). However, there is still a paucity of studies describing molecular features of breast tumors in East Africa (45, 46). Here, we studied mutational profiles in three ancestrally distinct patient groups, namely AA, EA, and Kenyan patients, and asked if these profiles show patient group differences or may associate with immune cell profiles or patients’ neighborhood environment. We found that the TMB was highest in the Kenyan samples, lower in AA, and lowest in breast tumors from EA patients. We further noted that mutations targeting *TBX3* were confined to EA patients and those targeting the *FBXW7* tumor suppressor to AA patients whereas mutations in the *ARID1A* gene were distinctively enriched among Kenyan patients.

*TBX3* mutations are putative driver mutations in breast cancer and usually lead to loss of function (47, 48). *TBX3* mutations were not among the recurrent mutations in a large Nigerian breast cancer cohort with 129 patients (11), in agreement with our finding that they are more common in EA patients. The *FBXW7* tumor suppressor gene is one of very few genes whose mutational frequency has previously been linked to ancestry (38). Our data show that these mutations are uncommon but largely restricted to AA patient with breast cancer where they promote oxidative phosphorylation and increased energy production, as our data suggest. This observation is consistent with the findings from a previous pan-cancer study (49). *ARID1A* mutations are present at high frequency in advanced endocrine therapy-resistant ER-positive breast tumors, as shown previously (50). Hence, their occurrence has clinical significance. Their presence will influence the decision about the therapy that should be given to patients with ER-positive breast cancer because an *ARID1A* mutation may render these patients insensitive to first-line endocrine therapy. Mechanistically, *ARID1A* mutations cause a loss of luminal identity and transdifferentiation to a basal-like phenotype that does not depend on ER activity (50). Kenyan patients with breast cancer present most commonly with ER-positive (60%–70%) breast cancer (13). Further investigations of ER-positive patients in both Kenya and surrounding countries should assess if *ARID1A* mutations indeed affect them at an increased frequency.

Triple-negative breast cancer impacts women of African ancestry more so than other women (51). The disease is a target of immune therapy (52, 53). Although it has been shown that the response to immune checkpoint inhibitors correlates with a high mutational burden in colon and non–small cell lung cancer, this relationship has not been established for breast cancer (54). Nevertheless, there is preliminary evidence that the combination of a PARP inhibitor with the immune checkpoint inhibitor, Pembrolizumab, may work best in patients with advanced triple-negative breast cancer with *BRCA* mutations (55). We investigated whether CIBERSORT-predicted immune cell profiles show variance in association with patient group and tumor mutation signatures. Overall, we did not find robust relationships. However, we observed a suggestive positive relationship between CD8 T-cell numbers and mutational signatures defined by AID/APOBEC cytidine deaminase activity–induced mutations, namely SBS2 and SBS13. This observation is consistent with previous findings analyzing Nigerian breast tumors. Here, the APOBEC mutational signature positively correlated with increased T-cell infiltration (11). We also found suggestive differences in the number of resting macrophages between the patient groups, with tumors from AA tumors harboring the highest numbers of these macrophages. We and others have previously reported that macrophage numbers might be increased in tumors of this patient group (19, 56), while others reported differences in CD8 T cells (57).

In the past, we and others reported that the *TP53* mutation frequency in breast tumors associates with patients’ socioeconomic status (58, 59), providing a rationale for our research approach to study the potential impact of the neighborhood environment on breast cancer biology. Our investigations were restricted to U.S. patients in our study as comparable data did not exist for the Kenyan patients. We found that neighborhood deprivation associates with disease characteristics and is a predictor of patient survival in a multivariable model. However, we did not find that it associates with mutational signatures of known molecular origin that are prevalent in human breast tumors. From this analysis, it does not appear that the neighborhood environment is a driver of the mutational landscape in these tumors; however, our study might have been underpowered to detect associations between NDI and these signatures. In an unrelated study, we showed that neighborhood deprivation associates with circulating immune-oncology markers and metastasis in patients with prostate cancer (32). Thus, it is possible that the NDI has a greater impact on the metastatic process by altering the immune environment in the circulation than it influences the immune biology of the primary tumor site.

Our study cohort included 9 male patients. There is limited information on the somatic mutational profile in breast tumors from male patients because these tumors are rare. Male breast cancers share many molecular features with female breast cancers although differences in mutation frequencies may exist, with male breast cancers having a lower mutational burden (60). As a limitation of our study, we did not include patients with breast cancer from West Africa as an additional comparison group. However, there have been reports on the mutational profile of breast tumors in Nigerian patients with breast cancer (11, 41), whereas data for East African patients have been missing. This is the reason why we focused on Kenyan patients with breast cancer. Findings from Pitt and colleagues (11) indicate that the mutational profile in tumors of Nigerian patients is similar to the profile in tumors of AA patients. In contrast, our study reports that ARID1A mutations may occur at an increased frequency in Kenyan patients.

In conclusion, our data show that mutational signatures may show distinct differences between patient groups of diverse race/ethnic background. Within the NCI-Maryland breast cancer cohort, we did not obtain evidence of a robust relationship between neighborhood deprivation and the occurrence of mutational signatures that commonly occur in breast tumors. This observation contrasts with previous findings from another study that revealed a relationship of neighborhood deprivation with circulating immune-oncology markers and lethal prostate cancer. Using a hypothesis-generating approach, analyzing 23 breast tumors from Kenyan patients, we discovered an overrepresentation of *ARID1A* gene mutations. These mutations are known to confer resistance to endocrine therapy. This intriguing finding should be followed up with a larger study.

## Supplementary Material

Supplementary Table 1 Patient characteristics for NCI-Maryland cohort.

Supplementary Table 2 Patient characteristics for Kenyan Cohort

Supplementary Table 3 Differentially expressed genes and pathway enrichment analysis comparing the 3 patient groups, EA, AA and Kenyan.

Supplementary Figure 1 Supplementary Figure 1 shows the effect of acquired FOXA1 somatic mutations on breast cancer survival.

Supplementary Figure 2 Supplementary Figure 2 shows the distribution of immune cell types in breast tumors.

Supplementary Figure 3 Supplementary Figure 3 shows mutational signatures in breast tumors and their association with somatic mutations in driver genes and immune cell signatures.

Supplementary Figure 4 Supplementary Figure 4 shows neighborhood deprivation as a determinant of breast cancer survival in the NCI-Maryland cohort.

Supplementary Figure 5 Supplementary Figure 5 shows neighborhood deprivation and its association with mutational signatures.
