# Supplementary material for: Population-specific Mutation Patterns in Breast Tumors from African American, European American, and Kenyan Patients
Source: Cancer Res Commun. 2023 Nov 7;3(11):2244–55. doi: 10.1158/2767-9764.CRC-23-0165 (PMC10629394; doi:10.1158/2767-9764.CRC-23-0165)
Supplement: Supplementary Table 1 — Patient characteristics for NCI-Maryland cohort. [file crc-23-0165-s01.docx]

| **Supplementary Table 1.** Patient characteristics for NCI-Maryland cohort | | | | |  |
| --- | --- | --- | --- | --- | --- |
| **Demographics** | **All  N=168** | | **AA  N=97 (57.7%)** | **EA N=69 (41)** | **Asian N=2 (1.3)** |
| Age |  | |  |  |  |
| Years, mean ± SD | 56.5±14.4 | | 56.8±14.3 | 56.6±14.4 | 39±2.8 |
| Sex |  | |  |  |  |
| Male | | 9 (5.4) | 2 (2.1) | 7 (10) | 0 |
| Female | 159 (94.6) | | 95 (97.9) | 62 (90) | 2 (100) |
| BMI |  | |  |  |  |
| kg/m^2^, mean ± SD | 30.6±7.2 | | 31.2±6.8 | 29.4±7.6 | 25.4±2.2 |
| Unknown | 17 (10.1) | | 10 (10.3) | 7 (10.1) | 0 |
| Hormone status |  | |  |  |  |
| Triple-negative^1^ | 34 (20) | | 23 (24) | 11 (16) | 0 |
| Estrogen receptor |  | |  |  |  |
| Positive | 105 (62.5) | | 56 (57.7) | 48 (69.6) | 1 (50) |
| Negative | 59 (35.1) | | 39 (40.2) | 19 (27.5) | 1 (50) |
| Unknown | 4 (2.4) | | 2 (2.1) | 2 (2.9) | 0 |
| HER2 receptor |  | |  |  |  |
| Positive | 27 (16.1) | | 18 (18.6) | 9 (13) | 0 |
| Negative | 114 (67.8) | | 62 (63.9) | 50 (72.5) | 2 (100) |
| Unknown | 27 (16.1) | | 17 (17.5) | 10 (14.5) | 0 |
| Stage |  | |  |  |  |
| I | 29 (17.2) | | 15 (15.5) | 14 (20.3) | 0 |
| II | 90 (53.6) | | 55 (56.7) | 33 (47.8) | 2 (100) |
| III | 39 (23.2) | | 22 (22.7) | 17 (24.6) | 0 |
| IV | 1 (0.6) | | 1 (1) | 0 | 0 |
| Phyllodes | 1 (0.6) | | 1 (1) | 0 | 0 |
| Unknown | 8 (4.8) | | 3 (3.1) | 5 (7.3) | 0 |
| Income |  | |  |  |  |
| Less than $15,000 | 25 (14.9) | | 21 (21.7) | 4 (5.8) | 0 |
| $15,000-$59,000 | 55 (32.7) | | 35 (36.1) | 20 (29) | 0 |
| Greater than $60,000 | 32 (19.1) | | 6 (6.2) | 24 (34.8) | 2 (100) |
| Unknown | 56 (33.3) | | 35 (36) | 21 (30.4) | 0 |
| Education |  | |  |  |  |
| Less than High School | 26 (15.5) | | 21 (21.7) | 5 (7.3) | 0 |
| High School | 42 (25) | | 21 (21.7) | 21 (30.4) | 0 |
| College/Technical School | 48 (28.6) | | 25 (25.7) | 22 (31.9) | 1 (50) |
| Graduate School | 12 (7.1) | | 3 (3.1) | 8 (11.6) | 1 (50) |
| Unknown | 40 (23.8) | | 27 (27.8) | 13 (18.8) | 0 |
| Diabetes |  | |  |  |  |
| No | 78 (46.4) | | 40 (41.2) | 38 (55.1) | 0 |
| Yes | 38 (22.6) | | 28 (28.9) | 10 (14.5) | 0 |
| Unknown | 52 (31) | | 29 (29.9) | 21 (30.4) | 2 (100) |
| Neoadjuvant Therapy |  | |  |  |  |
| No | 72 (42.8) | | 44 (45.4) | 28 (40.6) | 0 |
| Yes | 30 (17.9) | | 18 (18.6) | 12 (17.4) | 0 |
| Unknown | 66 (39.3) | | 35 (36) | 29 (42) | 2 (100) |
| Neighborhood Deprivation Index |  | |  |  |  |
| pc1990^2^ (Mean ± SD) | 1.716±3.2 | | 3.066±3.3 | -0.075±2.1 | -1.486±0.4 |
| pc2000^3^ (Mean ± SD) | 1.753±3.0 | | 3.053±2.8 | 0.019±2.2 | -1.061±0.9 |
| Unknown | 31 (18.5) | | 18 (18.6) | 13 (18.8%) | 0 |
| AA= African American, EA= European American, SD = standard deviation  ^1^Negative for estrogen, progesterone, and HER2 receptor expression  ^2^NDI with 1990 census tract level data  ^3^NDI with 2000 census tract level data   \|  \| \| --- \| |  | |  |  |  |
|  |  | |  |  |  |
|  |  | |  |  |  |
|  |  | |  |  |  |
|  |  | |  |  |  |
|  |  | |  |  |  |
|  |  | |  |  |  |
