# Supplementary material for: Population-specific Mutation Patterns in Breast Tumors from African American, European American, and Kenyan Patients
Source: Cancer Res Commun. 2023 Nov 7;3(11):2244–55. doi: 10.1158/2767-9764.CRC-23-0165 (PMC10629394; doi:10.1158/2767-9764.CRC-23-0165)
Supplement: Supplementary Table 2 — Patient characteristics for Kenyan Cohort [file crc-23-0165-s02.docx]

| **Supplementary Table 2.** Patient characteristics for Kenyan Cohort | |
| --- | --- |
| **Demographics** | **N = 23 (100%)** |
| Age, years (mean ± SD) | 48.8 ± 11.0 |
| BMI, kg/m^2^ (mean ± SD) | 32 ± 6.6 |
| Unknown, n = 11 |  |
| Tumor Grade |  |
| 1 | 4 (17.4) |
| 2 | 17 (73.9) |
| 3 | 2 (8.7) |
| Hormone status |  |
| Triple-negative^1^ | 3 (13) |
| Estrogen Receptor |  |
| Positive | 16 (70) |
| Negative | 7 (30) |
| Unknown | 0 |
| Her2 Receptor |  |
| Positive | 8 (34.8) |
| Negative | 13 (56.5) |
| Unknown | 2 (8.7) |
| Diabetes |  |
| No | 19 (82.6) |
| Yes | 2 (8.7) |
| Unknown | 2 (8.7) |
| \|  \| \| --- \| |  |
|  |  |
| SD = standard deviation, PR=progesterone. ^1^Negative for estrogen, progesterone, and HER2 receptor expression |  |
|  |  |
