# Supplementary material for: Population-specific Mutation Patterns in Breast Tumors from African American, European American, and Kenyan Patients
Source: Cancer Res Commun. 2023 Nov 7;3(11):2244–55. doi: 10.1158/2767-9764.CRC-23-0165 (PMC10629394; doi:10.1158/2767-9764.CRC-23-0165)
Supplement: Supplementary Figure 1 — shows the effect of acquired FOXA1 somatic mutations on breast cancer survival. [file crc-23-0165-s04.pdf]

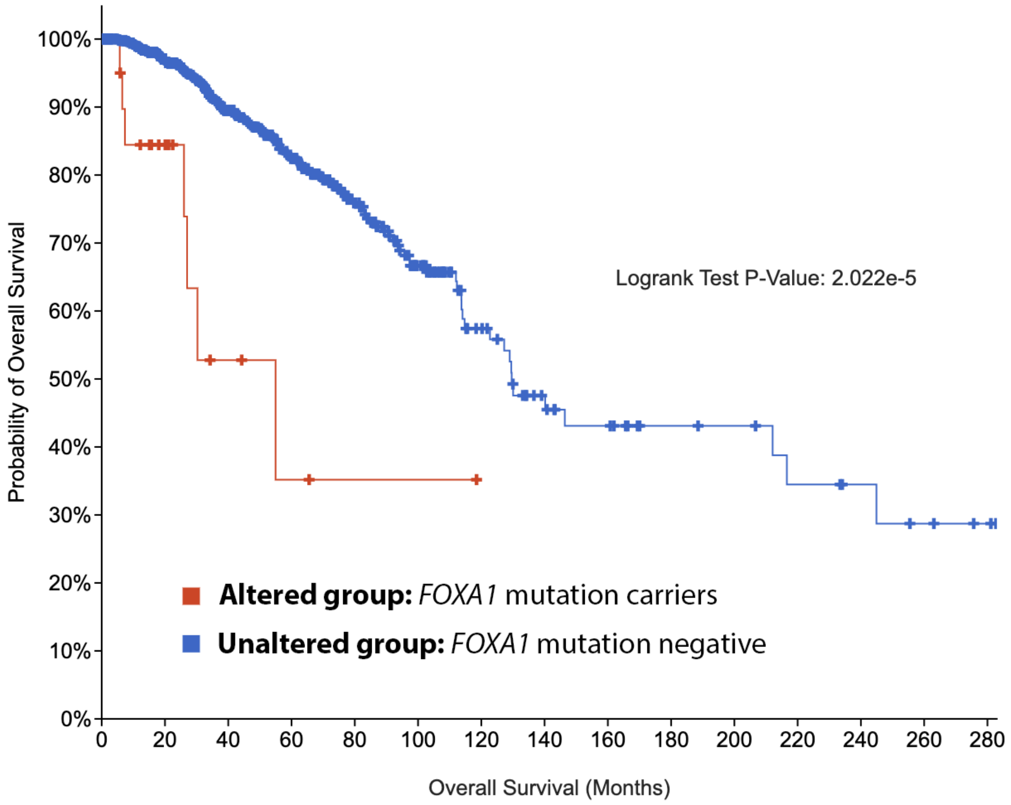

	Number of Cases, Total	Number of Events	Median Months Overall (95% CI)
Altered group	**23**	**7**	**54.96 (26.97 - NA)**
Unaltered group	**953**	**131**	**129.60 (114.72 - 244.91)**

Supplementary Figure 1. Effect of acquired FOXA1 somatic mutations on breast cancer survival. Mutations in the *FOXA1* gene have a deleterious effect on survival of breast cancer patients in the TCGA-Broad GDAC breast cancer cohort (https://gdac.broadinstitute.org/). Kaplan-Meier plot with log-rank test.
