# Supplementary material for: Population-specific Mutation Patterns in Breast Tumors from African American, European American, and Kenyan Patients
Source: Cancer Res Commun. 2023 Nov 7;3(11):2244–55. doi: 10.1158/2767-9764.CRC-23-0165 (PMC10629394; doi:10.1158/2767-9764.CRC-23-0165)
Supplement: Supplementary Figure 2 — shows the distribution of immune cell types in breast tumors. [file crc-23-0165-s05.pdf]

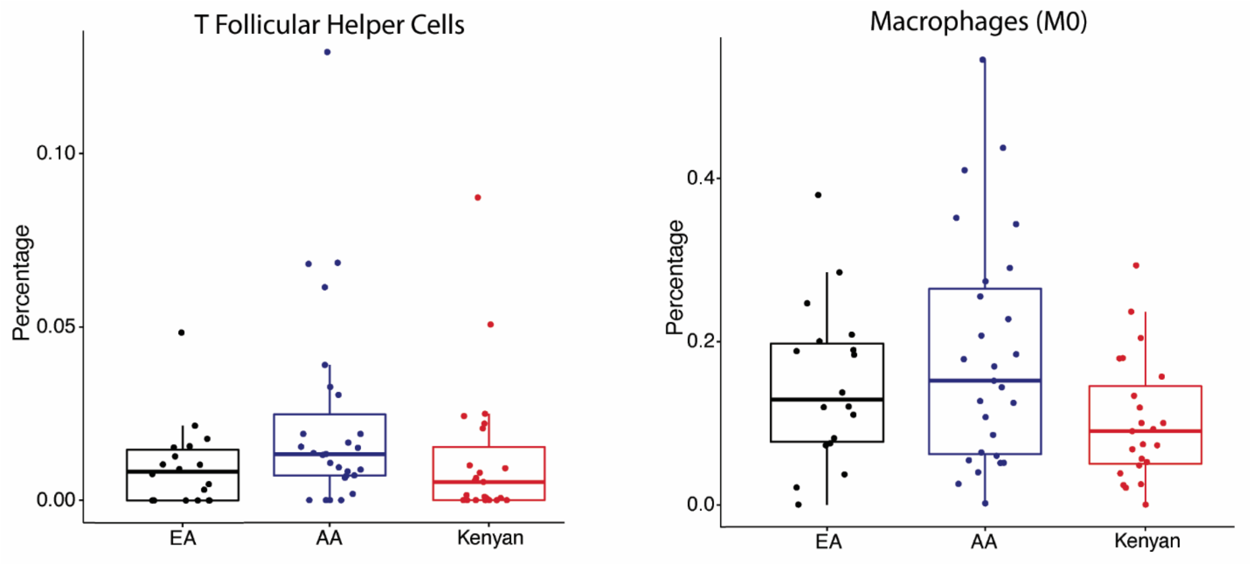

Supplementary Figure 2. Distribution of immune cell types in breast tumors. T follicular helper cells and uncommitted macrophages (M0) were elevated in breast tumors of AA but not Kenyan breast cancer patients when compared with EA patients. Relative abundance of immune cells in the tumor tissue is based on transcriptome data and the CIBERSORT algorithm.
