# Supplementary material for: Population-specific Mutation Patterns in Breast Tumors from African American, European American, and Kenyan Patients
Source: Cancer Res Commun. 2023 Nov 7;3(11):2244–55. doi: 10.1158/2767-9764.CRC-23-0165 (PMC10629394; doi:10.1158/2767-9764.CRC-23-0165)
Supplement: Supplementary Figure 3 — shows mutational signatures in breast tumors and their association with somatic mutations in driver genes and immune cell signatures. [file crc-23-0165-s06.pdf]

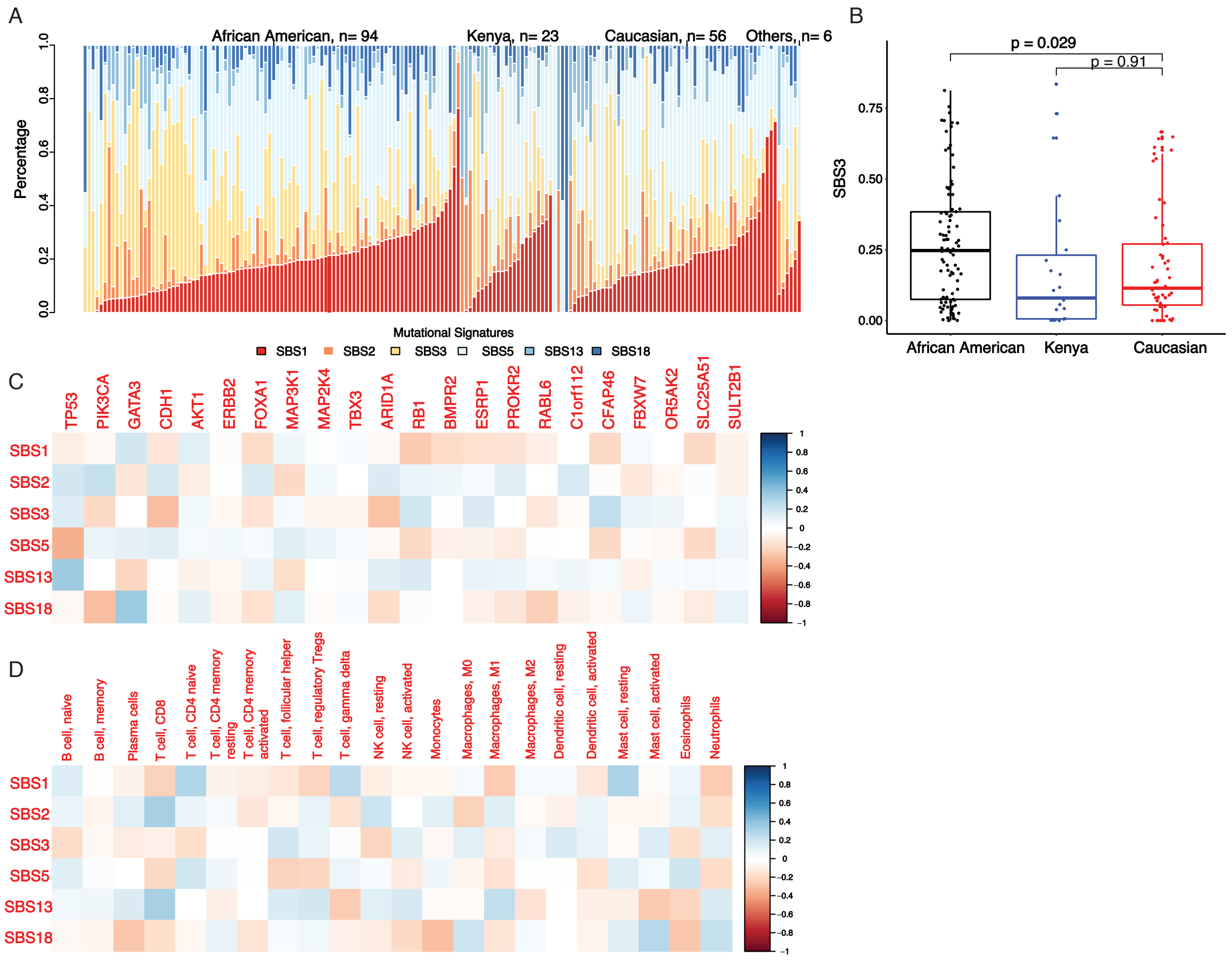

Supplementary Figure 3. Mutational signatures in breast tumors and their association with somatic mutations in driver genes and immune cell signatures. Sensitivity analysis generating the same data as shown in Figure 3 - but with male and Asian American patients being removed from the dataset. **(A)** Prevalence of mutational signatures from the COSMIC catalog (SBS1-3, 5, 13, 18) in breast tumors from African American (AA), Kenyan, and European American (EA) patients. Others includes 6 European American patients with self-reported Hispanic ethnicity. **(B)** Elevated presence of the SBS3 mutational signature in breast tumors of AA patients. Relative abundance scores for SBS3 in each tumor were compared between the 3 patient group patients (one-way ANOVA, $P < 0.05$). **(C)** Heatmap showing a correlation coefficient matrix for the relationship between the 6 COSMIC-based mutational signatures and somatic mutations in candidate driver genes in 179 breast tumors. **(D)** Heatmap showing a correlation coefficient matrix for the relationship between the mutational signatures and gene expression-based immune cell profiles of the tumors. Transcriptome data and the CIBERSORT algorithm were used to define the immune cell profiles.
