# Supplementary material for: Population-specific Mutation Patterns in Breast Tumors from African American, European American, and Kenyan Patients
Source: Cancer Res Commun. 2023 Nov 7;3(11):2244–55. doi: 10.1158/2767-9764.CRC-23-0165 (PMC10629394; doi:10.1158/2767-9764.CRC-23-0165)
Supplement: Supplementary Figure 4 — shows neighborhood deprivation as a determinant of breast cancer survival in the NCI-Maryland cohort. [file crc-23-0165-s07.pdf]

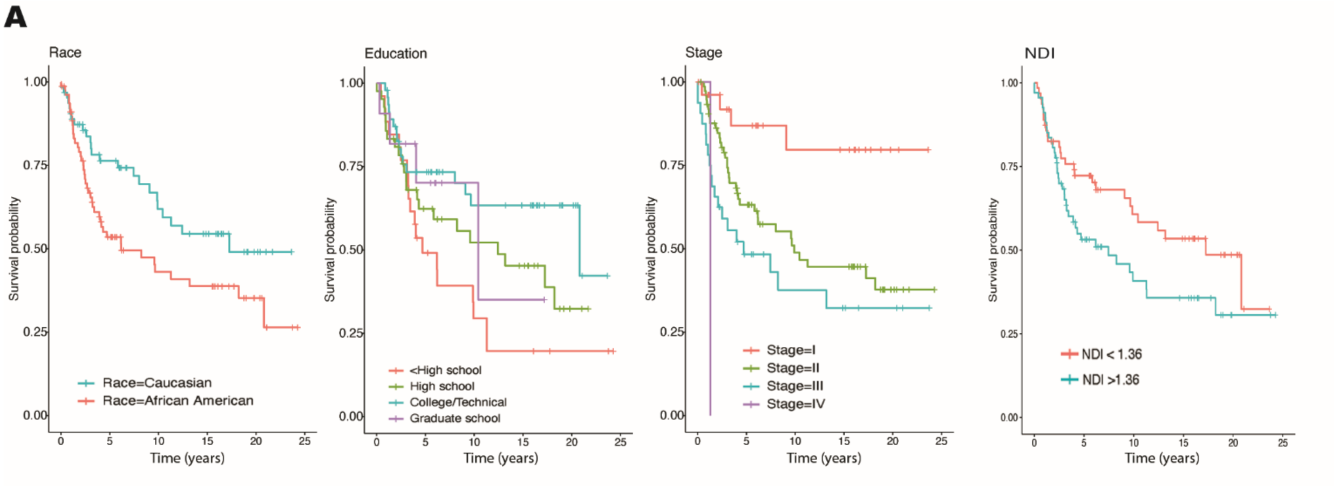

B

Overall Survival	HR	95% CI	P value
RACE	1.72	(1.04-2.84)	0.034
AGE	1.35	(1.12-1.63)	0.0015
BMI	1.01	(0.98-1.05)	0.48
ER	0.74	(0.45-1.22)	0.24
HER2	1.57	(0.87-2.84)	0.14
TNBC	1.19	(0.65-2.17)	0.57
STAGE	2.05	(1.39-3.02)	3E-04
INCOME	0.61	(0.41-0.9)	0.014
EDUCATION	0.69	(0.52-0.93)	0.014
DIABETES	1.97	(1.09-3.57)	0.026
NEOADJ	2.18	(1.22-3.89)	0.0087
NDI	1.09	(1.01-1.18)	0.025

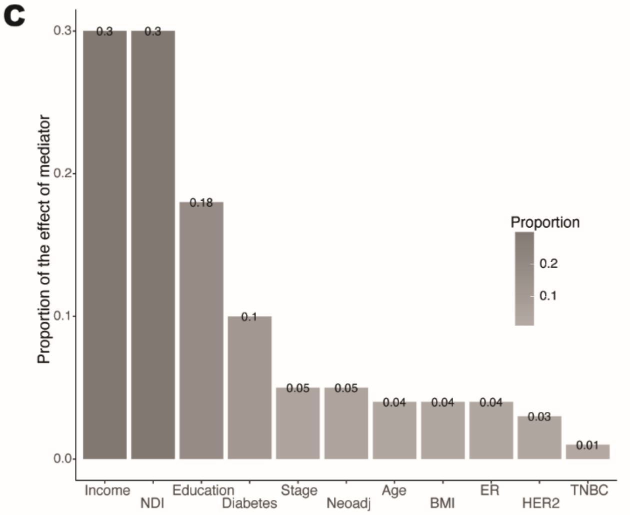

Supplementary Figure 4. Neighborhood deprivation as a determinant of breast cancer survival in the NCI-Maryland cohort. (A) Kaplan-Meier plots showing breast cancer survival probability by race/ethnicity, education, disease stage, and median dichotomized neighborhood deprivation index (NDI) in the NCI-Maryland cohort. Log-rank test: $P < 0.05$ each. **(B)** Predictors of breast cancer survival in the univariate survival analysis using Cox regression modeling. Self-reported race with European Americans as reference; income/education with low income/education as reference; age, BMI, and NDI were used as continuous variable. Red: significant associations. **(C)** Proportion of the effect of various mediators on breast cancer survival. Socioeconomic status and the neighborhood deprivation index (NDI) had the strongest independent effects on survival, followed by diabetes as a comorbidity and the disease stage. NDI for the survival analysis was derived from the 2000 census data.
