# Supplementary material for: Population-specific Mutation Patterns in Breast Tumors from African American, European American, and Kenyan Patients
Source: Cancer Res Commun. 2023 Nov 7;3(11):2244–55. doi: 10.1158/2767-9764.CRC-23-0165 (PMC10629394; doi:10.1158/2767-9764.CRC-23-0165)
Supplement: Supplementary Figure 5 — shows neighborhood deprivation and its association with mutational signatures. [file crc-23-0165-s08.pdf]

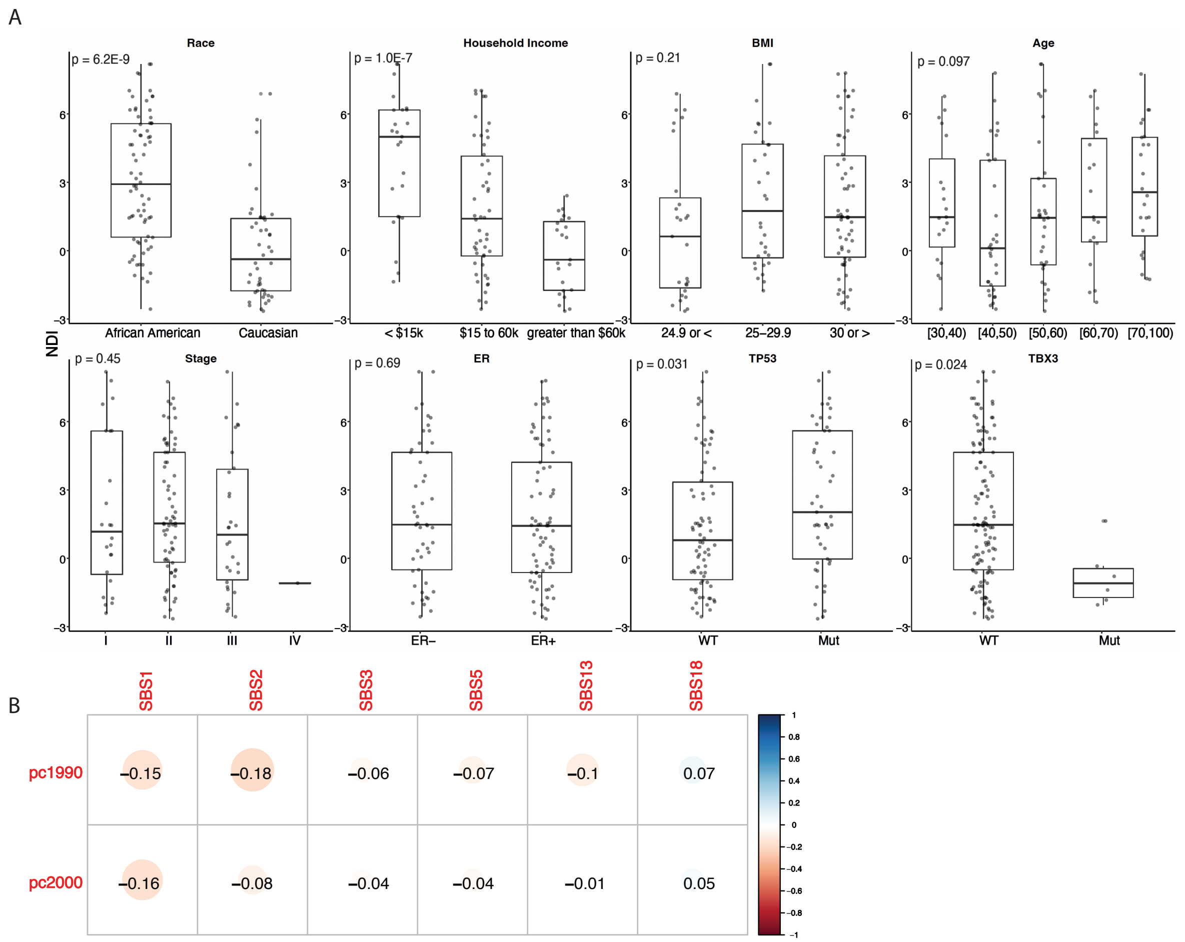

Supplementary Figure 5. Neighborhood deprivation and its association with mutational signatures. Sensitivity analysis generating the same data as shown in Figure 4 - but with male and Asian American patients being removed from the dataset. **(A)** Relationship of the neighborhood deprivation index (NDI) with race/ethnicity, income, body mass index (BMI), age, disease stage, tumor estrogen receptor status (ER), and tumor *TP53* and *TBX3* mutational status, in the NCI-Maryland breast cancer cohort. NDI for the analysis was obtained for each patient in the study using 2000 census data and correlated with patient or tumor characteristics. Significant associations with race, household income, and *TP53* and *TBX3* mutational status ($P < 0.05$ with t-test or one-way ANOVA) **(B)** Correlation matrix for the relationship of patients' 1990 and 2000 neighborhood deprivation indexes with 6 COSMIC-based mutational signatures in their tumors. NDI shows a moderate inverse correlation with the SBS1 and SBS2 signatures.
